# Sustained Recovery of Kidney Function in Patients with ESKD under Chronic Dialysis Treatment: Systematic Review and Meta-Analysis

**DOI:** 10.3390/nu15071595

**Published:** 2023-03-25

**Authors:** Carlo Garofalo, Chiara Ruotolo, Claudia Annoiato, Maria Elena Liberti, Roberto Minutolo, Luca De Nicola, Giuseppe Conte, Silvio Borrelli

**Affiliations:** Unit of Nephrology, Department of Advanced Medical and Surgical Sciences, University of Campania “Luigi Vanvitelli”, 80138 Naples, Italy

**Keywords:** recovery of kidney disease, end-stage kidney disease, dialysis

## Abstract

The prevalence of recovery of kidney function (RKF) in patients under maintenance dialysis is poorly defined mainly because of different definitions of RKF. Therefore, to gain more insights into the epidemiology of RKF, we performed a systematic review and meta-analysis of studies addressing the prevalence of sustained (at least for 30 days) RKF in patients under maintenance dialysis. Acute kidney injury (AKI) and RKF in the first 90 days of dialysis were the main exclusion criteria. Overall, 7 studies (10 cohorts) including 2,444,943 chronic dialysis patients (range: 430–1,900,595 patients) were meta-analyzed. The period of observation ranged from 4 to 43 years. The prevalence of RKF was 1.49% (95% C.I.:1.05–2.11; *p* < 0.001] with high heterogeneity I2: 99.8%, *p* < 0.001. The weighted mean dialysis vintage before RKF was 294 ± 165 days; RKF persisted for a weighted mean of 27.5 months. The percentage of RKF was higher in studies from the U.S. (1.96% [95% C.I.: 1.24–3.07]) as compared to other countries (1.04% [95%C.I.: 0.66–1.62]; *p* = 0.049). In conclusion, sustained RKF unrelated to AKI occurs in about 1.5% of patients under maintenance dialysis. On average, RKF patients discontinue chronic dialysis about ten months after starting treatment and live free of dialysis for more than two years. The higher prevalence of RKF reported in the U.S. versus other countries suggests a major role of country-specific policies for dialysis start.

## 1. Introduction

The sustained recovery of kidney function (RKF) is defined as the persistent discontinuation of kidney replacement treatment (KRT) in patients affected by end-stage kidney disease (ESKD) under maintenance dialysis [1]. Recent large surveys have reported that the prevalence of RKF is surprisingly high (4.5%) [2,3]. This finding is unexpected because (I) ESKD is an irreversible and permanent loss of renal function and (II) chronic dialysis worsens ischemic renal injury, therefore, hampering RKF [2].

The increased prevalence of RKF is, at least in part, due to the inclusion of patients who recovered kidney function before 90 days, that is patients with acute kidney injury (AKI). The temporal criterion is mandatory to define the chronicity of kidney failure and exclude patients with reversible AKI; on average, three weeks are required to recover from acute tubular necrosis [4]. Therefore, the inclusion of potentially reversible AKI may lead to an overestimation of the prevalence of sustained RKF and does not allow a correct interpretation of this phenomenon, since those patients might not be affected by a permanent loss of kidney failure [5,6].

The accurate assessment of sustained RKF and the consequent discontinuation of chronic dialysis is a critical decision because if, on the one hand, dialysis is a life-saving therapy, on the other hand, the survival and quality of life of patients treated by maintenance dialysis are dramatically poor compared to patients under conservative treatment [7]. Moreover, the sustained withdrawal of chronic dialysis has a significant social and economic impact, considering the high burden of dialysis on health resources [8].

Despite the great interest in this clinical issue, no meta-analysis has evaluated the actual impact of sustained RKF (no AKI-related) in patients affected by ESKD under chronic dialysis. Therefore, to fill this critical knowledge gap, we performed a systematic review and meta-analysis of studies to evaluate the epidemiology, that is, prevalence, temporal features, and correlates, of sustained and AKI-unrelated RKF in patients under maintenance dialysis.

## 2. Materials and Methods

The present review followed the Preferred Reporting Items for Systematic reviews and Meta-Analyses (PRISMA) guidelines [9]. We searched relevant articles published from inception until 30 November 2022 using PubMed, SCOPUS, and ISI Web of Science databases. The following Medical Subject Headings (MeSH) and text words were used: “renal function recovery” OR “kidney function recovery” OR “dialysis discontinuation” OR “renal recovery” AND “ESRF” OR “ESKD” OR “ESRD” OR “dialysis” OR “end-stage renal failure” OR “end-stage renal disease”. References of articles and reviews found in the research were further screened to identify additional studies.

### 2.1. Study Selection

Inclusion criteria were: (1) observational studies performed in chronic peritoneal dialysis (PD) or hemodialysis (HD) patients; (2) evaluating the percentage of sustained RKF as an outcome. We defined RKF as being sustained if discontinuing maintenance dialysis lasted for more than30 days.

We defined the prevalence of RKF excluding patients with ascertained AKI and when RKF occurred in the first 90 days from dialysis initiation. Accordingly, we excluded the studies if we could not distinguish between acute or chronic kidney failure.

Abstracts, letters to editors, commentaries, case series, and reviews were excluded from our review. Search strategies were limited to English-language studies, and when studies reported overlapping data, we chose those with complete information.

The titles and abstracts found with the search strategy were screened independently by two investigators (CG, CR). The full reports of potentially relevant studies were obtained, and each paper was reviewed using predefined eligibility criteria. Any discrepancy between the two authors on study eligibility was resolved through discussion. Data extraction was performed independently by two authors using standard data extraction forms.

### 2.2. Assessment of Risk of Bias

The Newcastle–Ottawa Scale (NOS) was used for quality assessment (Table 1) [10]. The risk of bias was assessed among the cohort studies by considering three items: (1) selection of participants (containing four domains); (2) comparability (one domain); and (3) outcome measure (containing three domains). Each domain was rated as “Yes”, “No”, or “Unclear”. Each quality domain was categorized as low risk for bias (Yes) when the study reported adequate data and met the criteria and high risk for bias (No) when the study did not meet the criteria for that quality domain. Studies that did not report data to assess quality were categorized as “Unclear” and thus potentially at high risk of bias. “Yes” was scored 1 and “No” or “Unclear” were scored “0”. A quality bar was plotted for each domain to examine the limitations of the studies. Studies of high quality were defined by scores > 5 points. Disagreements in the scores were addressed to achieve consensus between two reviewers.

### 2.3. Statistical Analysis

We quantified the inter-rater agreement for study selection and quality assessment. We performed a random-effect meta-analysis of the percentage of RKF extracted from selected studies. As additional analyses, we also performed a random effect meta-analysis of mean age, percentage of males, and peritoneal dialysis patients.

Extracted estimates were pooled in the meta-analysis. We assumed a conservative approach in pooling results by using a random-effects model, which allows for a variation of true effects across studies. We analyzed heterogeneity with the I^2^ statistic with 95% CI [11]. I^2^ values of 25%, 50%, and 75% correspond to cut-off points for low, moderate, and high degrees of heterogeneity. Sensitivity analyses were conducted to exclude that a study was exerting excessive influence on the heterogeneity [12]. Furthermore, univariate random-effects meta-regression and moderator analyses were performed to explore sources of heterogeneity when significant. Meta-regression was used to test the difference between moderators. Restricted maximum likelihood estimators were used to estimate model parameters [13]. We evaluated as continuous variable baseline age, the prevalence of male participants, the prevalence of diabetics, the prevalence of PD patients, dialysis vintage before RKF, observation period, and publication year. As categorical moderators, we evaluated the sample size of cohorts (<100,000 or ≥100,000 patients), the prevalence of patients treated by PD (> or <40%) and countries (U.S. vs. non-U.S. countries). We assessed potential predictors of sustained RKF expressed as odds ratio (OR) or hazard ratio (HR) if available at least in three studies. When we found OR as the main measure with an overall risk of outcome > 10%, we performed a sensitivity analysis to verify the risk of overestimating the association. The summary estimate was given as HR.

Funnel plot, Begg’s rank correlation test, and Egger’s linear regression were used to assess the publication bias [14]. A two-sided *p*-value < 0.05 was considered significant. The present systematic review and meta-analysis was registered in PROSPERO (CRD N. 42023394840)

Analyses were performed using PROMETA 2 (INTERNOVI, Cesena, Italy) and R Studio version 1.1442 (R Studio: Integrated development environment for R. Boston, MA, USA).

## 3. Results

**Figure 1:**
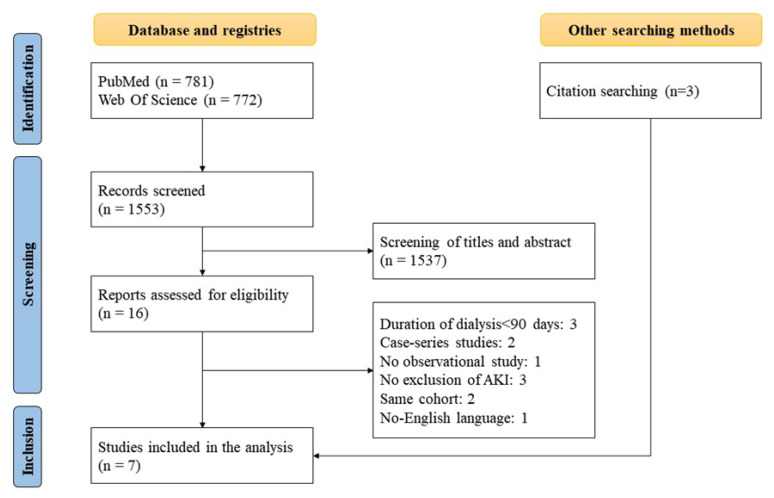
Algorithm of selection.

Based on the screening of titles and abstracts by databases, sixteen studies out of 1553 were initially considered. As illustrated in the flowchart (Figure 1), seven studies were included in the systematic review and meta-analysis [2,6,15,16,17,18,19]. The causes of exclusion were lack of information on AKI-related RKF, cohorts included in other selected studies (also if partially), case series studies, and no-English language (Figure 1). The agreement between the two reviewers for study selection was very good (Kappa = 0.843).

The study by Goldstein et al. [16] reported data from four different cohorts (New England and New Haven in PD and HD, respectively) with different follow up (Table 1); therefore, we could calculate the sustained RKF for each of four cohorts included in the study. Finally, we assessed 10 cohorts (6 from the U.S., 1 from Canada, 2 from Europe, and 1 from Australia/New Zealand) (Table 1).

Overall, 2,444,943 chronic dialysis patients (range: 430–1,900,595 patients) were included in the meta-analysis. The period of observation ranged from 4 to 43 years.

**Figure 2:**
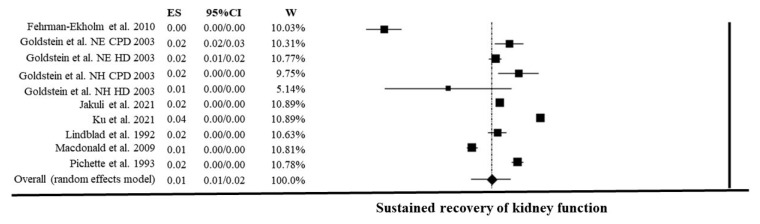
Random effect meta-analysis of recovery of kidney function percentage on 2,444,943 chronic dialysis patients [2,6,15,16,17,18,19].

The prevalence of RKF was 1.49% [95% C.I. (1.05–2.11); *p* < 0.001]. The effect size showed high heterogeneity I^2^: 99.8%, *p* < 0.001 (Figure 2). Sensitivity analysis did not find any significant effect of a single study on heterogeneity.

**Figure 3:**
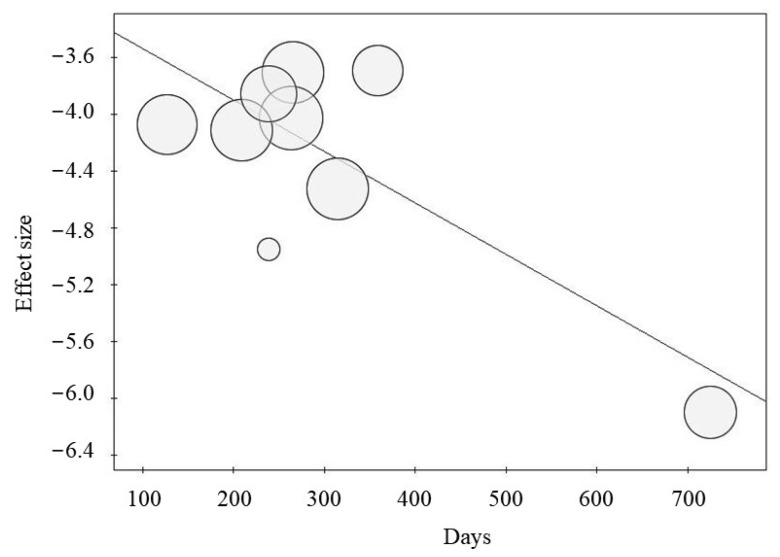
Meta-regression between dialysis vintage (days before RKF) and percentage of recovery of kidney function (*p* = 0.007).

Meta-regression analysis showed an inverse correlation between the percentage of RKF and the dialysis vintage before RKF (*p* = 0.007; Figure 3). Conversely, the mean age (*p* = 0.620), percentage of male gender (*p* = 0.586), percentage of peritoneal dialysis (*p* = 0.562), and prevalence of diabetes (*p* = 0.240) did not correlate with RKF. Similarly, the percentage of RKF was not correlated with publication year (*p* = 0.950) and the duration of observation (*p* = 0.710).

**Figure 4:**
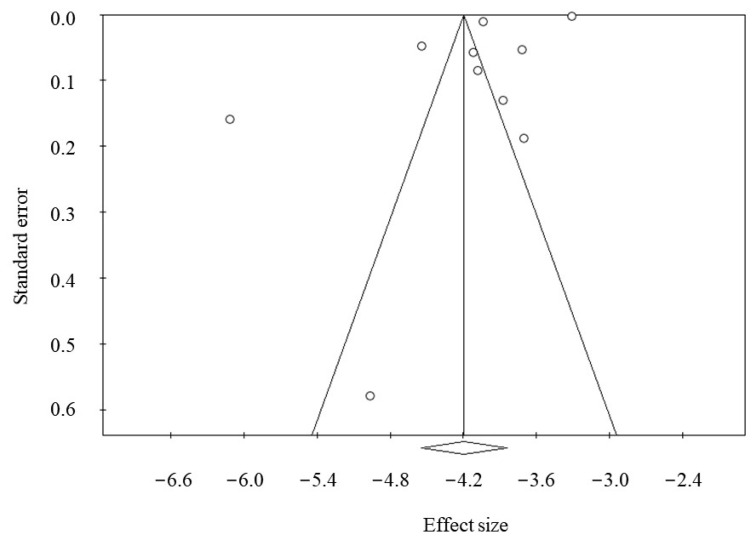
Funnel plot of publication bias (Egger’s test: *p* = 0.086; Begg and Mazumdar’s test: *p* = 0.245).

Moderator analysis showed that country influenced heterogeneity; in particular, the percentage of RKF was greater in studies from the U.S. (1.96 % [95%CI, 1.24–3.07]) as compared to other countries (1.04 % [95% CI, 0.66–1.62]; *p* = 0.049). No significant association was found with the sample size (*p* = 0.109). Furthermore, we did not find any significant effect of percentage of patients treated by PD (> vs. <40% PD use) on the RKF prevalence (*p* = 0.276). Except for gender, the main potential factors associated with sustained RKF (e.g., age, race, primary kidney disease, residual diuresis, eGFR at dialysis initiation) were not available for the meta-analysis. We found female gender was not significantly associated with a sustained RKF (HR: 1.06 [ 95%C.I.: 0.94–1.20]; *p* = 0.301). Publication bias was not found as testified by the Funnel plot (Figure 4) and Egger (*p* = 0.086) and Begg’s test (*p* = 0.245).

### Features of RKF Patients

The n-weighted mean dialysis vintage before RKF was 294 ± 165 days. The duration of RKF was evaluated in four studies for a total of 505 patients; RKF persisted for an n-weighted mean of 27.5 months. Random effect meta-analysis showed a mean age (available in 6 studies) of 64.3 years (95%CI, 61.8–66.8), a prevalence of males (available in 6 studies) of 59% (95% C.I.: 58–60%), and a percentage of PD (available in 8 studies) of 50% (95% C.I.: 27–73%). The prevalence of primary kidney diseases in RKF patients showed that diabetes was prevalent (33%) in only one study [2], whereas in other studies, the prevalence was lower or absent. Notably, in the other cohorts, the prevalence of systemic disease such as vasculitis, systemic lupus erythematosus (SLE), scleroderma, amyloidosis, myeloma and Hemolytic Uremic Syndrome (HUS) were the prevalent primary kidney diseases in patients who recovered kidney function (Table 2).

## 4. Discussion

This meta-analysis shows that the overall percentage of sustained RKF after excluding reversible causes of kidney failure is 1.49% in a population of about 2.5 million chronic dialysis patients. Sustained RKF occurs after a mean of 10 months from the beginning of chronic dialysis (up to 24 months), although the probability of a sustained RKF decreases for longer dialysis vintage (Figure 3). Notably, dialysis-independent life persists on average for over two years (27.5 months).

The choice to select AKI-unrelated and sustained RKF allows us to achieve a more realistic estimation of this phenomenon. As expected, the exclusion of reversible causes showed a remarkable reduction in the prevalence of sustained RKF in patients under maintenance dialysis with ascertained ESKD than reported in recent large surveys [2,3]. Nonetheless, the high heterogeneity of the prevalence of RKF (I^2^ = 99.8%), ranging from 0.22% to 3.55% in our study (Table 2), raises the question of the factors that are associated with recovery of a sufficient renal function to discontinue maintenance dialysis in patients with an apparent irreversible and progressive renal disease.

Due to the extreme heterogeneity of the demographic and clinical features of patients who recovered kidney function and, consequently, discontinued maintenance dialysis, we could not draw a profile of the factors associated with RKF. We found that the unique factor associated with a significant difference in the estimation of prevalence of RKF was the categorization according to the country study (U.S studies vs. non-U.S. studies). Specifically, the percentage of RKF is significantly greater in the studies performed in the U.S. compared to other countries (Canada, Europe, and Australia/New Zealand). The interpretation of this finding is not intuitive; however, we can speculate that the heterogeneity of RKF is due to the different policies for dialysis starting used in each country. Although the eGFR level at the beginning of dialysis was not registered in the studies of our meta-analysis, the U.S. dialysis registry reports that patients started chronic dialysis with a GFR >10 mL/min/1.73 m^2^ [20], which is a value higher than that observed in patients starting dialysis in Europe and Australia/New Zealand (<7 mL/min/1.73 m^2^) [18,21]. Accordingly, Mac Donald et al. reported that higher probability of RKF was associated with a higher eGFR at dialysis initiation [18].

To support our hypothesis, we found that in the U.S. survey collecting incident dialysis patients in the last two decades [2], primary kidney diseases generally inducing irreversible and permanent loss of kidney function (e.g., diabetes, hypertension, and ADPKD) accounted for over 50% of the patients who discontinued chronic dialysis (Table 2). Conversely, in non-U.S. countries, RKF has been reported in renovascular and obstructive diseases, which may recover renal function after the removal of the specific causes (renal artery stenosis and calculi, respectively). Furthermore, ESKD may be one of the manifestations of systemic diseases such as vasculitis, SLE, scleroderma, amyloidosis, HUS, and paraproteinemia diseases, which may show a response to therapy even several months after the beginning of maintenance dialysis. Indeed, in these systemic diseases, patients may recover kidney function because of the administration of immunosuppressants or chemotherapies after the start of chronic dialysis [22,23,24,25]. Moreover, it is interesting that if we consider only the potentially reversible primary kidney diseases, the RKF percentage in the U.S. registry decreases from 3.55 to 1.29%, which is in line with other countries. Accordingly, the analysis of the predictors of RKF in a subgroup of U.S. patients starting dialysis between 2012 and 2016 showed that the higher likelihood of recovering renal function was associated with the primary kidney diseases classified as “other” (aHR:3.37 [95%C.I.: 3.17–3.57]), “unknown” (aHR:2.02 [95%C.I.: 1.81–2.26]), and “missing cause” (aHR: 7.41; [95%C.I.: 4.03–13.62]), which likely included potentially reversible systemic diseases (e.g., vasculitis, HUS, etc.) [3]. These findings are consistent with European [6] and Swedish dialysis [19] registries, which reported that systemic diseases were common causes of sustained RKF (Table 2).

European dialysis registry did not provide information about geographical differences among the countries on the prevalence of sustained RKF [6]. Nonetheless, in the Swedish registry [19], the prevalence of RKF was 0.22%, which is lower than in other countries [2,6,18]; however, this lower prevalence is due to the stricter inclusion criterion to select sustained RKF used in that study, which considered RKF only after 12 months of dialysis at variance with other studies using 90 days as the threshold level for RKF definition. This observation is consistent with the findings of the meta-regression performed in this study (Figure 3) showing an inverse correlation between likelihood of RKF and dialysis vintage (before RKF).

Notably, except for gender, we could not evaluate the predictors of RKF by meta-analysis because the measures of RKF risk associated with the main potential predictors (e.g., age, diabetes, dialysis technique) were not comparable among the studies. Nonetheless, we explored the potential role of those factors on the heterogeneity of our estimation by analysis of moderators and meta-regression.

Our meta-analysis showed that the female gender was not significantly associated with the risk of sustained RKF. These findings were consistent with the contrasting results reported in the dialysis registries [2,6,18]. Indeed, women had a higher likelihood of sustained RKF in the European registry [6], while RKF risk was lower for females in the U.S. dialysis registry [2,3]. No difference between males and females was found in the Australia and New Zealand Dialysis and Transplant (ANZDATA) registry [18].

Furthermore, our meta-regression exploring the relationship between RKF and age showed that the prevalence of RKF was not associated with the mean age of cohorts. Again, this result may depend on the heterogeneity of the results on the association between age and the probability of sustained RKF reported in the studies. In the U.S. dialysis registry, the likelihood of RKF was higher in younger patients [2,3], whereas the likelihood of RKF was higher in older patients in the European dialysis registry [6]; no correlation with age was found in the ANZDATA registry [18].

Remarkably, the meta-analysis showed that the discontinuation of chronic dialysis was unrelated to the dialytic technique (PD vs. HD). Indeed, both meta-regression and the analysis of moderators showed no correlation between the percentage of peritoneal dialysis use and the prevalence of sustained RKF. This is an interesting point because peritoneal dialysis could be considered protective for residual kidney function and may potentially favor the recovery of kidney function. Accordingly, Goldstein et al. reported a higher incidence of RKF in patients treated by peritoneal dialysis [16]. However, the ANZDATA registry showed the that the possible advantage of PD disappeared after adjustment for sex, gender and race [18], and U.S. and European registries showed a higher likelihood in patients treated by hemodialysis [2,3,6].

Overall, our findings support the hypothesis that the higher percentage of sustained RKF observed in the U.S. is related to an earlier beginning of dialysis than in other countries. Considering that the IDEAL trial did not demonstrate any survival advantage to start chronic dialysis at a higher GFR level (9.0 mL/min/1.73 m^2^) than those patients who started dialysis at lower eGFR level (7.2 mL/min/1.73 m^2^) [26], we can hypothesize that a number of those patients initiating dialysis with higher GFR may be treated for a long term with conservative care providing that uremic complications are strictly controlled [27]. Therefore, decisions on dialysis start maybe more based on the trend of eGFR change and the whole clinical picture rather than merely being dictated by the eGFR level. This approach should theoretically prevent the anticipated start of dialysis and the RKF phenomenon as well [28]. Similarly, no criteria to discontinue chronic dialysis was recognized in the studies evaluating sustained RKF. The critical decision to suspend chronic lifesaving KRT remains based on the opinion and the experience of the nephrologists that may explain the high heterogeneity in the studies evaluating this phenomenon.

Nevertheless, our results highlight that free-dialysis ESKD management may persist for more than two years; therefore, the periodic monitoring of residual GFR in non-anuric dialysis patients should be encouraged. In this regard, it is also important to underline that a recent study showed that a higher ultrafiltration rate and intra-dialytic hypotension during dialysis vintage were associated with a lower likelihood of RKF [3], suggesting the need to implement strategies to maintain residual kidney function as long as possible in chronic dialysis patients [29]. Unfortunately, the lack of information about residual diuresis and urine output in the patients under maintenance dialysis who recovered kidney function prevents drawing any causal inferences on the relationship between residual eGFR and the likelihood of RKF.

The retrospective nature of all studies included in the meta-analysis does not allow us to provide a definitive answer on this issue. Therefore, we cannot exclude that patients might recover function because of the improvement of treatment as observed in CKD patients referred to nephrologists. However, this occurrence becomes uncommon in more advanced CKD stages associated with diabetes and ADPKD [30].

This meta-analysis has some limitations: (1) the studies of the meta-analysis are all retrospective, and the quality of each study is modest; (2) there is no information about Asian and African patients, which limits the generalizability of our findings; (3) we cannot perform a meta-analysis for the main RKF predictors because the adjusted risks in the single studies were not comparable; (4) the lack of shared criteria evaluating the discontinuation of chronic dialysis in the patients who recovered kidney function.

## 5. Conclusions

This meta-analysis provides evidence that AKI-unrelated and sustained RKF is infrequent as it occurs in about 1.5% of uremic patients under chronic maintenance dialysis. Furthermore, the higher prevalence reported in the U.S. is probably related to the beginning of dialysis at a higher GFR level than in other countries. This policy for dialysis start is likely more susceptible to discontinuation even in patients affected by diabetic kidney disease and ADPKD, which are considered causes of irreversible and permanent loss of kidney function, because of improved control of complications in patients with a significant residual GFR. Finally, these data encourage the strict monitoring (and preservation) of residual renal function in the first months of dialysis treatment and underline the need for a common and shared policy for evaluating sustained RKF and, consequently, withdrawing chronic dialysis.

## Figures and Tables

**Table 1:** The Newcastle–Ottawa Scale (NOS) was used for quality assessment.

Author/ Country/Publication Year	Sample Size (N)	Calendar Period (year)	Age (Years)	Males (%)	Diabetes (%)	PD (%)	RKF N/%	Days before RKF	RKF Duration (Months)	Quality Score
Lindblad/US/1992	8288	1981–1988	n.a.	n.a.	n.a.	100	138/1.67	126	n.a.	4
Pichette/Canada/1993	14,318	1981–1989	n.a.	n.a.	n.a.	n.a.	243/1.7	n.a.	n.a.	4
Goldstein (NE CPD)/US/2003	2924	1979–1999	n.a.	n.a.	n.a.	100	60/2.05	240	n.a.	4
Goldstein (NE HD)/US/2003	19,032	1993–1999	n.a.	n.a.	n.a.	0	305/1.60	210	n.a.	4
Goldstein (NH CPD)/US/2003	1200	1993–1998	n.a.	n.a.	n.a.	100	29/2.42	360	37.0	4
Goldstein (NH HD)/US/2003	430	1993–1998	n.a.	n.a.	n.a.	0	3/0.70	240	n.a.	4
Macdonald/Australia -New Zealand/2009	39,570	1963–2006	53.4	59.4	20.5	40.2	420/1.06	316	24.5	4
Fehrman-Ekholm/Sweden/2010	17,590	1991–2008	n.a.	n.a.	19.3	26.0	39/0.22	726	47.2	5
Jakulj/Europe/2019	440,996	1997–2016	67.9	63.0	24.1	18.0	5465/1.23	265	n.a.	4
Ku/US/2021	1,900,595	1996–2015	63.0	55.6	46.8	7.79	67541/3.55	n.a.	n.a.	4

Abbreviations: PD, peritoneal dialysis; RKF, recovery of kidney function; n.a., not available.

**Table 2:** Prevalence of primary kidney disease (%) in patients with the recovery of kidney function (*N* = 74243).

Ref.	DKD	Hypertension	Glomerular	ADPKD	IN	Others	Unknown	AD-RVD	Vasculitis -SLE–Scleroderma	Amyloidosis- Myeloma-	HUS
[15]	n.a.	n.a.	n.a.	n.a.	n.a.	n.a.	n.a.	n.a.	n.a.	n.a.	n.a.
[17]	n.a.	n.a.	n.a.	n.a.	n.a.	n.a.	n.a.	n.a.	n.a.	n.a.	n.a.
[16]	0	0	13	0	0	63	n.a.	8–15	n.a.	n.a.	n.a.
[16]	0	0	17	0	6	51	n.a.	15–14	n.a.–n.a.–2	2–n.a.	0
[16]	0	0	10	0	7	27	n.a.	38–10	7	0–0	0
[16]	0	0	0	0	0	33	0	33–33	0	0–0	0
[18]	n.a.	n.a.	n.a.	n.a.	n.a.	n.a.	n.a.	n.a.	n.a.	n.a.	0
[19]	2.6	0	10.3	0	2.6	10.3	17.9	5.1–20.5	7.7–5.1–5.1	2.6–0	10.3
[6]	11.0	9.7	11.8	0.3	9.9	18.2	22.8	2.9–n.a.	5.4–n.a.–0.9	n.a.–5.4	1.7
[2]	32.7	28.1	12.9	2.6	n.a.	16.5	7.0	n.a.	n.a.	n.a.	n.a.

Abbreviations: DKD: Diabetic Kidney Disease; ADPKD; Autosomal Dominant Polycystic Kidney Disease; IN: Interstitial Nephropathy; AD: Atheroembolic disease; RVD: Reno-vascular disease; SLE: Systemic Lupus Erythematosus; HUS: Hemolytic Uremic Syndrome. n.a.: not available.
